# Prevalence of Dyslipidemia among Elementary School Children in Birjand, East of Iran, 2012

**Published:** 2016-01-13

**Authors:** Fatemeh Taheri, Toba Kazemi, Bita Bijari, Kokab Namakin, Mahmoud Zardast, Tayyebeh Chahkandi

**Affiliations:** *Birjand Atherosclerosis and Coronary Artery Research Center, Birjand University of Medical Sciences, Birjand, Iran.*

**Keywords:** *Dyslipidemias*, *Child*, *Prevalence*, *Iran*

## Abstract

**Background: **Various studies have indicated that dyslipidemia starts in childhood. There is a relationship between the concentration of blood lipids and atherosclerosis. In this study, we assessed the prevalence of dyslipidemia in elementary school children.

**Methods:** This cross-sectional study was performed on 1,626 (882 girls and 744 boys) elementary school children aged between 6 and 11 years in Birjand in 2012. Samples were selected through multistage random sampling. The lipid profiles (i.e., cholesterol, triglyceride, high-density lipoprotein cholesterol [HDL-C], low-density lipoprotein cholesterol [LDL-C]) of the subjects were measured after 12 hours’ fasting. The definition of dyslipidemia was based on the criteria of The American Children Academia in 2011.

**Results:** Dyslipidemia (at least one abnormal level of a serum lipid) was detected in 31% of the children (31.3% of the girls vs. 30.6% of the boys). Total cholesterol was ≥ 200 mg/dL in 13.4% of the children, LDL-C was ≥ 130 mg/dL in 8.5%, HDL-C was < 40 mg/dL in 11.3%, and triglyceride was ≥ 130 mg/dL in 15.3%. The prevalence of hypertriglyceridemia was significantly higher in the females than in the males (16.3% in the girls vs. 13.8% in the boys; p value = 0.01) - while hypercholesterolemia (12.2% in the girls vs. 14.9% in the boys; p value = 0.16), high LDL-C (8.5% in the girls vs. 8.5% in the boys; p value = 0.05), and low HDL-C (12.7% in the girls vs. 9.5% in the boys; p value = 0.1) were not significantly different between the two sexes.

**Conclusion:** The prevalence of dyslipidemia was high in the school children in Birjand and, thus, requires preventive measures.

## Introduction

Cardiovascular diseases (CVDs) are one of the main causes of morbidity and mortality worldwide, and atherosclerosis is the principal underlying cause of CVDs in adults. Various studies have proved that there is a relationship between the concentration of blood lipids and atherosclerosis lesions.^1^ Other studies have indicated that atherosclerosis begins during childhood and that the concentration of serum lipids has a relationship with the occurrence of atherosclerotic lesions.^2^^-^^5^ In other words, although CVDs are not observed in childhood, cardiac risk factors such as dyslipidemia are present in children and they remain silent until adulthood.^3^^, ^^4^

Dyslipidemia is a disorder in lipoprotein metabolism that results in such abnormalities as high total cholesterol (TC), high low-density lipoprotein cholesterol (LDL-C), low high-density lipoprotein cholesterol (HDL-C), and high triglycerides (TG). Serum lipid levels in children are correlated with sex, race, and age.^6^ In many countries, due to the decrease in the consumption of traditional foods and increase in the consumption of fatty and fast foods as a result of industrialization and urbanization, the levels of blood lipids are on the rise.^7^^-^^12^

In adults, correcting serum lipids decreases CVD risks. Studies conducted on adults and retrospective studies on children have revealed that immediate and appropriate interventions to correct dyslipidemia in children slow down the process of atherosclerosis and postpone CVD onset. Research has demonstrated that in a few developing countries, the prevalence of dyslipidemia has decreased.^13^

Similar to many developing countries, CVDs in Iran are on the increase and, as such, are deemed a major health concern.^14^^, ^^15^ Given the high prevalence of dyslipidemia in South Khorasan Province^16^ and its relationship with childhood dyslipidemia, we sought to determine the levels of serum lipids and prevalence of dyslipidemia in elementary school children in the Iranian city of Birjand in 2012.

## Methods

The present study was conducted on 1700 elementary school students in Birjand. Samples were chosen through multistage cluster random sampling. First, Birjand was divided into five regions based on socioeconomic levels. Afterward, the elementary schools were identified in each region. The number of students in all the schools was obtained from the city’s health center. Ten elementary schools for each sex were chosen considering the distribution of the schools in the different districts of the city. Then, some students were selected from each class with respect to the population of each school and its ratio to the total number of students in that class. Based on the above procedure, 1700 students (6 to 11 years old) were chosen, and a demographic questionnaire together with a consent form was sent to each student’s parents. The parents were asked to fill out the questionnaire and the consent form and return them to the school. In this stage, 1626 questionnaires were completed and returned. According to the study plan, the students were referred to a diagnostic clinic and a 12-hour fasting blood sample was taken from each for testing. The obtained samples were immediately centrifuged. Via an enzymatic procedure using German Rosh kits and Biochemical AutoAnalyzer Prestige 24i (Japan), lipid levels were determined in each student. 

The National Heart, Lung, and Blood Institute (NHLBI) panel definition (2011) of dyslipidemia was used in our study.^2^ These cutoff points delineate lipid values as acceptable, borderline, and abnormal (Table 1).

**Table 1:** Definition of dyslipidemia in children and adolescence (mg/dL)

Category	Acceptable	Borderline High	High
TC mg/dL	< 170	170-199	≥ 200
LDL mg/dL	< 110	110-129	≥ 130
Non-HDL mg/dL	< 120	120-144	≥ 145
HDL mg/dL	> 45	45-40	≤ 40
TG mg/dL			
< 9 y 10-19 y	< 75 < 90	75-99 90-129	≥ 100 ≥ 130

TC, Total cholesterol; LDL, Low-density lipoprotein; HDL, High-density lipoprotein; TG, Triglyceride

For the statistical analyses, the statistical software SPSS version 15.0 for Windows (SPSS Inc., Chicago, IL) was used. Prevalence was described in percentages, while α ≤ 0.05 was taken as the significant level.

## Results

The study population comprised 1626 students, aged between 6 and 11 years. There were 882 (54.2%) girls and 744 (45.8%) boys at a mean age of 9.1 ± 1.4 years. Most of the students were aged between 9 and 10 (21.8%) and between 8 and 9 (20.2%) years.

Table 2 presents the mean of serum lipids. According to the table, the mean HDL-C and TG levels were significantly higher in the girls than in the boys, whereas the mean levels of the other lipids were not significantly different between the two sexes.

**Figure 1:**
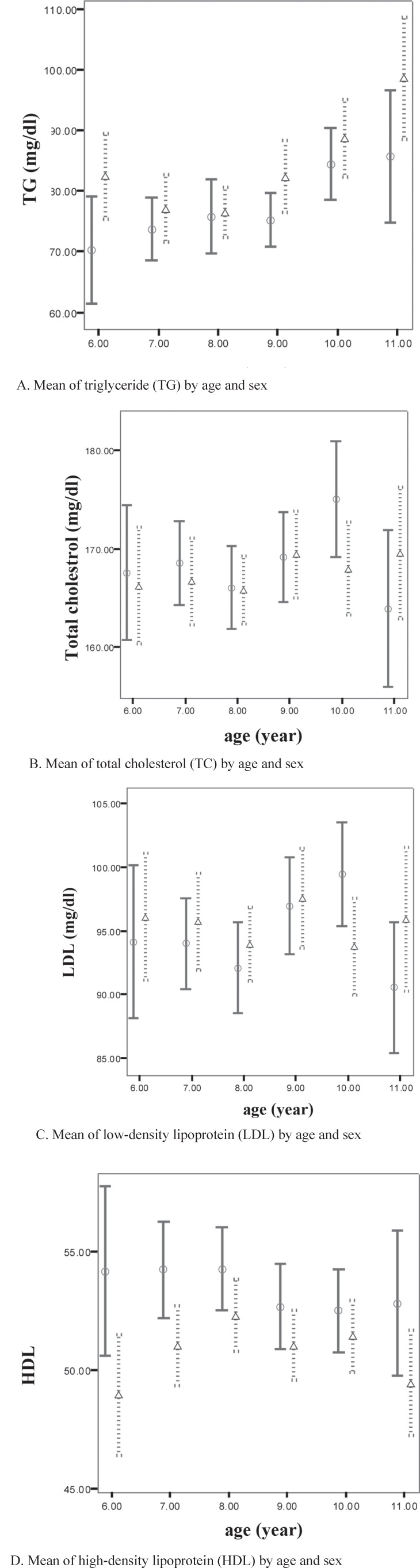
Mean of Lipid and lipoprotein levels by sex and age in our subjects

Figure 1 introduces the mean of serum lipids in terms of sex. Only the mean of TG significantly increased with age. The highest TG mean (92.96 ± 45.05 mg/dL) was diagnosed in the 11-year-old subjects, while the least TG mean (75.39 ± 31.38 mg/dL) was observed in the 7-year-old children (p value < 0.001).

Table 3 depicts the prevalence of dyslipidemia. In 31% (n, 502) of the students, there was at least one abnormal lipid disorder; and in 69.2% (n, 1121) of the students, the level of at least one serum lipid was higher than normal (borderline or abnormal).

The most frequent lipid disorder was hypercholesterolemia (43.2%) (TC ≥ 170 mg/dL), followed by hypertriglyceridemia. (In 39.2% of the children, TC was ≥ 100 mg/dL.) Moreover, HDL-C was low (< 45 mg/dL) in 24.7% of the study population. The least frequent disorder was LDL-C (≥ 100 mg/dL in 23.4%). The prevalence of hypertriglyceridemia was significantly higher in the girls (42.2% in the girls vs. 35.2% in the boys). The levels of the other lipids were not significantly different between the two sexes.

**Table 2:** Comparison of the mean serum lipids in our subjects by sex

	All (n=1626)	Males (n=744)		Females (n=882)		P Value
	Mean (SD)	Mean (SD)	Range	Mean (SD)	Range	
TC	168.4 (28.7)	169.1 (29.6)	(52 to 433)	167.7 (27.8)	(64 to 344)	0.332
LDL-C	95.3 (23.3)	95.1 (23)	(36 to 164)	95.5 (23.5)	(28 to 197)	0.791
HDL-C	52.1 (10.8)	53.3 (11.4)	(21 to 98)	51.1 (10.2)	(26 to 85)	< 0.001
						
TG	81.3 (37.2)	78.3 (35.9)	(16 to 361)	83.8 (38.1)	(26 to 346)	0.003

TC, Total cholesterol; LDL-C, Low-density lipoprotein cholesterol; HDL-C, High-density lipoprotein cholesterol; TG, Triglyceride

**Table 3:** Comparison of the amount and percentage of serum lipids levels (acceptable, borderline, and abnormal) in our study based on sex

Criteria		All	Males	Females	P Value
TC					0.164
Acceptable		924 (56.8)	406 (54.6)	518 (58.6)	
Borderline		485 (29.8)	228 (30.0)	257 (29.2)	
Abnormal		217 (13.4)	110 (14.9)	107 (12.2)	
					
LDL-C					0.503
Acceptable		1244 (76.6)	561 (75.5)	683 (77.6)	
Borderline		241 (14.9)	110 (16.0)	122 (13.9)	
Abnormal		139 (8.5)	63 (8.5)	76 (8.5)	
					
HDL-C					0.101
Acceptable		1225 (75.3)	576 (77.4)	649 (73.6)	
Borderline		218 (13.4)	97 (13.0)	121 (13.7)	
Abnormal		183 (11.3)	71 (9.5)	112 (12.7)	
					
TG					0.016
Acceptable		992 (61.0)	482 (64.7)	510 (57.8)	
Borderline		388 (23.9)	160 (21.4)	228 (25.9)	
Abnormal		246 (15.3)	103 (13.8)	113 (16.3)	

*Data are presented as n (%)

TC, Total cholesterol; LDL-C, Low-density lipoprotein cholesterol; HDL-C, High-density lipoprotein cholesterol; TG, Triglyceride

## Discussion

Based on the findings of the present study, the mean levels of TC, LDL-C, HDL-C, and TG were 168, 95, 52, and 81 mg/dL, respectively. The mean HDL-C level was lower in the female students, while the mean TC level was higher in the girls. The mean levels of the other lipids were not significantly different between the two sexes.

According to a study on children between 6 and 12 years old in the Iranian province of South Khorasan in 2006, the mean levels of TC, LDL-C, HDL-C, and TG were 152 mg/dL (150 mg/dL in the boys vs. 154 mg/dL in the girls), 90 mg/dL (87 mg/dL in the boys vs. 93 mg/dL in the girls), 44 mg/dL, and 87 mg/dL - respectively. Additionally, the mean levels of TC and LDL-C were significantly higher in the girls.^17^ A comparison between the findings of the present study and those of the South Khorasan Province study reveals an increase in the mean levels of all lipids. A study carried out in South Korea reported an increase in the mean levels of lipids in children. (For instance, the mean LDL-C level rose from 89.3 to 96.5 mg/dL between 1998 and 2001.)^18^

A study on children aged between 10 and 14 years in Tehran between 2005 and 2008 reported that the mean TC level was 162 mg/dL in the boys and 160 mg/dL in the girls, the mean LDL level was 95 mg/dL in the boys and 104 mg/dL mg/dL in the girls, the mean HDL-C was 46.8 mg/dL in the boys and 43.7 mg/dL in the girls, and the mean TG level was 82 mg/dL in the boys and 95 mg/dL in the girls.^19^ A comparison between the results of the present study and those of the aforementioned study indicates that the mean levels of TG and HDL-C in Birjand are higher, while the mean levels of LDL-C and TC are not different between the two cities. However, the South Khorasan Province study in 2006 showed that the mean levels of HDL-C, LDL-C, TG, and TC in Birjand were lower than those in Tehran.^17^^, ^^19^

According to the National Health and Nutrition Examination Survey (NHANES) III in the United States on subjects aged between 6 and 15 years, the mean levels of TC, LDL-C, HDL-C, and TC in boys and girls were 158-172, 164-169, 88-94, and 87-96, respectively.^20^ Nonetheless in a recent study on young subjects in the United States between 1988 and 1994 and between 2007 and 2010, a favorable trend in serum lipid concentrations was observed.^21^

A study on children aged between 10 and 11 years in South Korea reported that the mean levels of TC were 166 mg/dL in the girls and 168 mg/dL in the boys, the mean levels of LDL-C were 96 mg/dL in the girls and 99 mg/dL in the boys, the mean levels of HDL-C were 51 mg/dL in the girls and 52 mg/dL in the boys, and the mean levels of TG were 93 mg/dL in the girls and 82 mg/dL in the boys.^22^

The findings of a study on 4,824 students aged between 6 and 18 years from 6 Iranian cities (Tabriz, Rasht, Gorgan, Mashhad, Yazd, and Tehran) as well as the findings of a few previous studies in Iran showed that Iranian adolescents rank higher in the TG percentile and lower in the HDL-C percentile than their peers in the United States and many other countries.^10^^, ^^11^ Another study performed in the Iranian city of Isfahan on 2000 students and another one in South Khorasan Province reported that the TC, TG, and LDL-C percentiles were higher than those reported in the United States, as presented in *Lipid* Research Clinics. (LRC), while the HDL-C percentile was lower.^11^^, ^^17^^, ^^21^

A comparison between the mean levels of lipids in the present study and those in other studies reveals that the levels among our study population were higher than those reported in South Khorasan Province in 2006. A comparison between children in Tehran and Birjand shows that the children from Birjand had higher levels of TC and HDL-C, equal level of LDL-C, and lower level of TG. A comparison with South Korean adolescents shows that in Birjand, the mean TC and LDL-C levels were higher and the mean HDL-C level was the same. Compared with South Korean subjects aged between 10 and 11 years, the children from Birjand had almost the same TC level, slightly higher LDL-C level, lower TG level, and equal HDL-C level.^17^^-^^19^

Our results revealed a significant finding. According to previous studies in Iran, the mean TG level of Iranian children is higher and their mean HDL-C is lower than those reported in the United States. Additionally, most of the studies in Iran have reported a high prevalence rate of low HDL-C level in Iranian children and adolescents.^19^ In contrast, we found that the mean TG level was lower and the mean HDL-C level was slightly higher than those reported in the United States. Further studies are required to shed sufficient light on the reason for these differences; nonetheless, the possible role of ecological-racial varieties and local food products should not be discounted.

The present study showed that the total prevalence of dyslipidemia was 31%. The lipid disorders - in order of prevalence - were hypercholesterolemia (43.2%), hypertriglyceridemia (39.2%), low HDL-C (24.7%), and high LDL-C (23.4%). Different studies have reported various prevalence rates of dyslipidemia. Thus, in addition to racial, genetic, and environmental factors causing differences in the lipid levels of children in various areas, different definitions of dyslipidemia should be taken into account. A study conducted in the Iranian capital, Tehran, on children between 7 and 10 years of age in 2001 found that the TC level of 31% of the subjects was at a borderline level and that of 17% was over 200 mg/dL. The authors also reported that LDL-C was high in 25%, HDL-C was low in 9%, and TG was high in 7% of their study population.^12^ In Tehran between 2005 and 2008, hypercholesterolemia was found in 8.5% of the males and 11.8% of the females, high LDL-C in 8.8% of the males and 10.1% of the females, low HDL-C in 25.2% of the males and 35.5% of the females, and hypertriglyceridemia in 4% of the males and 5.7% of the females.^19^

A study conducted on 7620 elementary school children in 23 provinces of Iran reported that 45.7% of the study population had dyslipidemia, the most prevalent manifestation of which was low HDL-C (24.8%) - followed by increased TG (24.5%) and hypercholesterolemia (6.4%). In addition, high LDL-C (6.3%) was the least frequent lipid disorder.

A study on children aged between 10 and 18 years and adolescents in South Korea revealed that - according to the National Cholesterol Education Program (NCEP) criteria-19.7% of the subjects had at least one type of dyslipidemia. Also, the mean HDL-C level in 14.5% of the study population was below 40 mg/dL, 6.5% had hypercholesterolemia, LDL-C in 4.7% was over 130 mg/dL, and TG in 10.1% was over 150 mg/dL. Additionally, the levels of hypercholesterolemia and high LDL-C were higher in the females than in the males. The values in the South Korean children and adolescents were compared with those of American white children in the NHANES in the United States between 1999 and 2006. It was found that hypercholesterolemia was less frequent in the South Korean subjects (6.5% vs. 9.3%), the mean LDL-C level was similar, high LDL-C was less prevalent in the South Koreans (4.7% vs. 7.7%), hypertriglyceridemia was more frequent in the American whites, and low HDL-C was more frequent in the South Koreans. The total prevalence of dyslipidemia in South Korea and the United States was close (19.7% vs. 20.3%). In South Korea, the prevalence of hypercholesterolemia and high LDL-C was higher in the females, which was discordant with the findings in the United States. This prevalence has been observed in some other Asian countries such as Iran^12^ and Korea.^18^

In the previous study in the South Khorasan Province, the most prevalent dyslipidemia reported were low HDL-C (14.1%), hypertriglyceridemia (5.4%), high LDL-C (4.1%), and hypercholesterolemia (3%). 

According to a study in Tehran in 2001, high LDL-C and high cholesterol were the first and the second most prevalent manifestations of dyslipidemia.^12^ Between 2005 and 2008, low HDL-C was the most prevalent dyslipidemia in the same city.^19^ A research in Saudi Arabia on 1390 children aged between 9 and 12 years found that high TG was the most prevalent lipid disorder.^23^ A study in Bangkok showed prevalence rates of 40% and 5.4% for high cholesterol and high LDL-C, correspondingly.^24^

The findings of the present study are different from the results of the previous study in the South Khorasan Province in terms of the most prevalent demonstration of dyslipidemia, while they correspond with the findings in the Bangkok study. Apropos sex, the present study found that hypertriglyceridemia was more prevalent in the girls, but the other forms of dyslipidemia were not significantly different between the two sexes. Nevertheless, the previous study in the South Khorasan Province reported that high LDL-C and low HDL-C were more prevalent in the girls.^17^

A comparison between the findings of the present study and those of the study in the South Khorasan Province in 2006 reveals an increase in TC, HDL-C, and HDL-C as well as a decrease in TG - indicating the rise of dyslipidemia among the children of the province. It should be noted that the previous study did not determine the borderline levels of lipids but mentioned the prevalence of abnormal conditions. A comparison between the findings of the two studies reveals a more-than-fourfold increase in hypercholesterolemia, an approximately threefold rise in high TG, and an approximately twofold increase in LDL-C. Furthermore, the prevalence of low HDL-C has decreased according to the findings of the current study.^17^

Other studies have shown that the mean cholesterol level in Iranian children and in the children of many other countries is on the rise.^11^ Hypercholesterolemia among the children in Birjand, similar to their peers in many developing communities, can be due to changes in lifestyle such as decreased consumption of traditional foods in consequence of industrialization and urbanization. These phenomena often increase the consumption of fast and fatty foods, which are decisive factors in increasing lipidemia and obesity. Indeed, various studies have reported the growing trend of obesity and abdominal obesity in children - particularly in developing countries.^25^^, ^^26^ Shortage of mobility because of the substitution of past active games with computer games and TV watching during recent decades may have been effective in the onset of dyslipidemia.

The major limitation to the present cross-sectional study is that we did not assess the influence of some factors such as nutrition, physical activity, and dietary on dyslipidemia. 

## Conclusion

Dyslipidemia had a high prevalence among the elementary school children in the Iranian city of Birjand. Screening the levels of lipids in children, especially those at risk, and accurate follow-up of those with dyslipidemia are suggested. 

## Notes:

***This paper should be cited as:*** Taheri F, Kazemi T, Bijari B, Namakin K, Zardast M, Chahkandi T. Prevalence of Dyslipidemia among Elementary School Children in Birjand, East of Iran, 2012. J Teh Univ Heart Ctr 2016;11(1):15-20.
